# Free Open Access Medical Education (FOAM) Resources in a Team-Based Learning Educational Series

**DOI:** 10.5811/westjem.2017.11.35091

**Published:** 2017-12-13

**Authors:** Timothy Fallon, Tania D. Strout

**Affiliations:** Tufts University School of Medicine, Maine Medical Center, Department of Emergency Medicine, Portland, Maine

## Abstract

Although Free Open Access Medical Education (FOAM) has become popular within emergency medicine, concerns exist regarding its role in resident education. We sought to develop an educational intervention whereby residents could review FOAM resources while maintaining faculty oversight. We created a novel curriculum pairing FOAM from the Academic Life in Emergence Medicine (ALiEM) Approved Instructional Resources (Air) series with a team-based learning (TBL) format. Residents have an opportunity to engage with FOAM in a structured setting with faculty input on possible practice changes. This series has been well-received by residents and appears to have increased engagement with core content material. Qualitative feedback from residents on this series has been positive and we believe this is the first described use of TBL in emergency medicine.

## BACKGROUND

Free Open Access Medical Education (FOAM) has rapidly expanded within emergency medicine (EM). Since 2013, 183 blogs and podcasts have focused on EM; however, a 2016 review identified content corresponding to only 71.5% of the required core content in EM as defined by the American Board of Emergency Medicine’s *Model of the Clinical Practice of Emergency Medicine*.^1^ In advocating FOAM in resident education, Nickson argues that physicians must develop the ability to evaluate the relevance of specific content and suggests that a flipped-classroom model “guards against FOAM resources being misunderstood by learners if they do not have sufficient base knowledge or clinical experience to appreciate the nuances.”^2^

We hypothesized that pairing FOAM with a team-based learning (TBL) approach would allow us to integrate these resources while maintaining faculty oversight and improving engagement. The TBL technique has been used widely in medical education. A systematic review in June 2016 identified 118 references to TBL in health professions education; however, 47% included medical students and only 6% related to residents.^3^ Poeppelman completed a review of the use of TBL in graduate medical education in 2016 and found no reported use within EM training.^4^

## OBJECTIVES

We sought to develop a structured educational intervention whereby residents could review curated FOAM that covered core content and innovation and integrate this knowledge with a TBL session. The intervention was designed to be easily integrated into our current didactic schedule with no need for additional faculty resources.

## CURRICULAR DESIGN

We created this series by pairing Academic Life in Emergence Medicine (ALiEM) Approved Instructional Resources (Air) series content to a TBL session. ALiEM Air has been used by 125 EM residencies and over 1,200 residents as of June 2017.^5^ While FOAM can be subject to concerns regarding quality, ALiEM was selected because of its clearly defined peer-review process.^6^ Two to four FOAM resources are selected to highlight 1–2 specific topics within a content module. We anticipate that residents will spend one hour in advanced preparation. A 50-minute TBL session includes both an individual and team quiz.^7^ The quiz is a combination of the ALiEMU quiz and questions created by the faculty to highlight key points.

During the team component, each question is discussed and each team’s pooled answer is presented. Residents have an opportunity to discuss the content within the team as well as with the larger group, sharing knowledge and experience. The faculty leads a guided discussion based on the responses. Cumulative team scoring for the year encourages adequate preparation. To date, sessions covering cardiology, trauma, EM procedures, and HEENT were created to coordinate with the content areas of the month during which they were scheduled. The figure provides a flow diagram for the asynchronous and classroom components of TBL.

## IMPACT/EFFECTIVENESS

This didactic series using FOAM and TBL has been well received by residents and appears to have increased engagement with the content. The TBL format provides opportunities for senior residents to teach junior colleagues. Although formal efficacy evaluation has not been conducted, resident qualitative feedback has been positive. Feedback was solicited following each session using our conference feedback mechanism as well as annually during our program curriculum review. Following the first four sessions, residents provided 32 unique comments.

The following are representative comments: “good discussion, great to have reading structure outside of conference;” “very engaging;” “FOAM materials were high quality… I found myself reading beyond the assigned topics;” and “it’s great working collaboratively with the upper level residents.” Some residents found the repetitive nature of individual and team-based questions was not helpful and some found the overall classroom to be too loud. In response, we introduced a phone app to complete quizzing and the format was altered slightly to allow each team to present the teaching points from one resource after the individual quiz and team discussion. When polled following this change, nine of 17 respondents preferred the new format with team-based teaching.

We have demonstrated an educational innovation through the use of FOAM paired to a TBL approach that requires no additional classroom time or faculty resources. We anticipate that this format will enable residents to critically appraise FOAM resources in a setting that allows for faculty input and oversight. This is the first report of the use of TBL within an EM residency of which we are aware. Future evaluation should focus on the educational effectiveness of this model and implementation in graduate medical education where this technique is relatively underrepresented in the literature.^3^

## Figures and Tables

**Figure:**
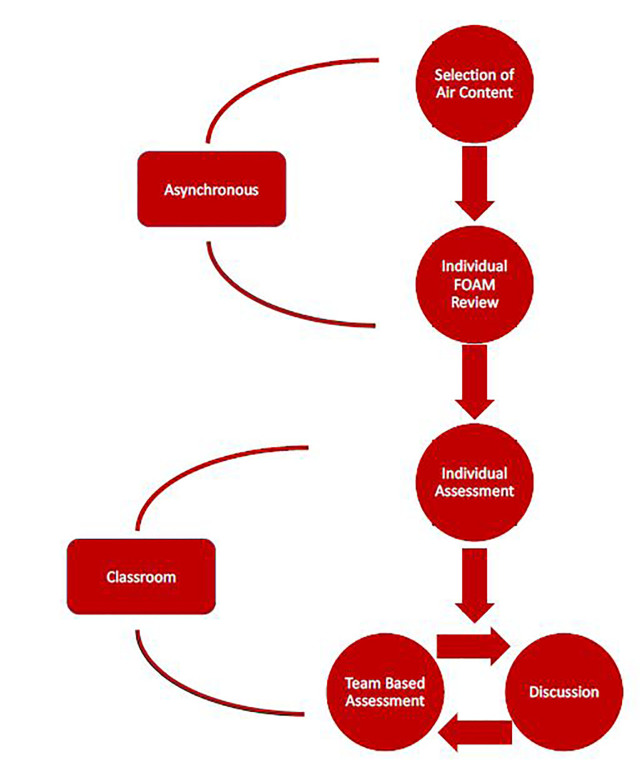
Curricular structure. *FOAM*, Free Open Access Medical Education.
